# Improving the Prognostic Ability through Better Use of Standard Clinical Data - The Nottingham Prognostic Index as an Example

**DOI:** 10.1371/journal.pone.0149977

**Published:** 2016-03-03

**Authors:** Klaus-Jürgen Winzer, Anika Buchholz, Martin Schumacher, Willi Sauerbrei

**Affiliations:** 1 Charité–Universitätsmedizin Berlin, Klinik für Gynäkologie mit Brustzentrum, Berlin, Germany; 2 Universitätsklinikum Freiburg, Institut für Medizinische Biometrie und Statistik, Department für Medizinische Biometrie und Medizinische Informatik, Freiburg, Germany; 3 Universitätsklinikum Hamburg-Eppendorf, Institut für Medizinische Biometrie und Epidemiologie, Hamburg, Germany; INRS, CANADA

## Abstract

**Background:**

Prognostic factors and prognostic models play a key role in medical research and patient management. The Nottingham Prognostic Index (NPI) is a well-established prognostic classification scheme for patients with breast cancer. In a very simple way, it combines the information from tumor size, lymph node stage and tumor grade. For the resulting index cutpoints are proposed to classify it into three to six groups with different prognosis. As not all prognostic information from the three and other standard factors is used, we will consider improvement of the prognostic ability using suitable analysis approaches.

**Methods and Findings:**

Reanalyzing overall survival data of 1560 patients from a clinical database by using multivariable fractional polynomials and further modern statistical methods we illustrate suitable multivariable modelling and methods to derive and assess the prognostic ability of an index. Using a REMARK type profile we summarize relevant steps of the analysis. Adding the information from hormonal receptor status and using the full information from the three NPI components, specifically concerning the number of positive lymph nodes, an extended NPI with improved prognostic ability is derived.

**Conclusions:**

The prognostic ability of even one of the best established prognostic index in medicine can be improved by using suitable statistical methodology to extract the full information from standard clinical data. This extended version of the NPI can serve as a benchmark to assess the added value of new information, ranging from a new single clinical marker to a derived index from omics data. An established benchmark would also help to harmonize the statistical analyses of such studies and protect against the propagation of many false promises concerning the prognostic value of new measurements. Statistical methods used are generally available and can be used for similar analyses in other diseases.

## Introduction

Understanding and improving the prognosis of patients with a disease or health condition is a priority in clinical research and practice. In the PROGnosis RESearch Strategy (PROGRESS) series a framework to improve research of interrelated prognosis themes has been proposed [1–4]. Two of the key topics are the role of prognostic factors and prognostic models. Since the beginning of the century, much of the research has been focused on issues related to personalized or stratified medicine with the assessment of genetic markers and analyses of high dimensional data as the challenge for researchers in many disciplines. A substantial part of such studies investigates issues for patients with cancer, breast cancer thereby being the disease considered most often [5–11]. Unfortunately, most of the results from the very large number of individual studies have not been validated and the number of clinically useful markers is pitifully small [12–14]. There are many potential pitfalls inherent in the complex process of successfully developing and validating a marker from omics data [15].

For some years it has been discussed to improve prediction rules through the integration of clinical and gene expression data [5,16–20]. However, applying combined prediction rules at a broader level would cause difficulties in many (smaller) centers and increase costs. Obviously, to be cost effective the predictive value of a combined prediction rule would need to be much larger than the predictive value of rules based on some generally available clinical measurements. In other terms, the added value of the genetic information would need to be ‘substantial’. Yet, assessing the added predictive value of genetic data to clinical data is far from trivial. Boulesteix and Sauerbrei [21] critically discuss various approaches for the construction of combined prediction rules and review procedures that assess and validate the added predictive value. Obviously, adding predictive value from genetic information to a ‘good’ clinical model is much more difficult than adding value to a ‘less good’ clinical model. Knowing about difficulties in using a combined model in practice, it follows that one may try to ‘optimize’ the predictive value from a model based on clinical data. The use of a combined predictor would only be sensible if the genetic information adds substantial predictive value to such an ‘optimized’ clinical predictor.

Notation in this area of research is confusing. Despite of using terms like ‘prediction’ and ‘added predictive value’ we will not consider the role of ‘predictive factors’, a term popular in cancer research where it usually implies that a factor is relevant for treatment decision. Such aspects require additional investigations (for example analysis of subgroups or investigation for an interaction between treatment and a factor) which will not be considered here [4]. This paper concentrates on prognostic factors and prognostic models [2,3]. The latter is a formal combination of multiple prognostic factors and the term predictor is popular and will be used here.

A number of prognostic models are published for many diseases, but for practical use the situation is often confusing. For example, there are over 100 models for prostate cancer [22] and for chronic kidney disease Collins et al [23] identified 14 prediction models featuring 43 different risk predictors. However, in breast cancer the situation seems to be better than for most other areas. Similar to the other cases, many classification schemes have been proposed, most of which did not survive validation [24,25]. But more than 30 years ago, the Nottingham Prognostic Index (NPI) was proposed, has been sufficiently validated, is well accepted and also used in clinical practice [26]. The three components lymph node stage, grade and tumor size are combined to a continuous index. Originally two and later up to five cutpoints were proposed to create three to six categories with different prognosis [26,27]. The simplicity of the NPI is certainly an important criterion for its clinical relevance, but it also implies that some prognostic information may not be used to improve the NPI. Applying elaborated statistical methods–we explain the key ideas and cite relevant references but do not provide all details—to a clinical registry data set [28] we extract the full information of the data and derive a modified version of the NPI with improved prognostic ability. We will illustrate that the continuous variables tumor size and number of positive nodes exhibit more information and will investigate whether another six standard variables carry prognostic information in addition to the NPI. With overall survival as the outcome of interest we will use the Cox model [29] for analysis. An often neglected but relevant issue is the verification of the model assumptions. We will check whether important assumptions of the Cox proportional hazard model are acceptable or whether modifications are required. A fundamental assumption is the proportionality of the hazard functions [30]. As known for many other diseases, prognostic effects varying in time have also been shown for clinical [31,32] and for genetic data [7,33] in breast cancer research. Nevertheless, even in top-ranking cancer journals it is rather an exception to report the check of this important assumption [34,35] and we speculate that suitable checks have often not been conducted. There is a severe danger of misspecification and models derived may be (partly) wrong with a potential consequence of incorrect medical decisions. However, poor reporting prevents to assess whether a relevant check of the model was not done or just not reported. To improve on this aspect several reporting guidelines, of which TRIPOD [36] and REMARK [37] are most relevant for the type of study discussed here, have been developed. They aim to Enhancing the QUAlity and Transparency Of health Research (EQUATOR network).

As discussed in the PROGRESS series [1–4] it is important having a suitable prognostic index with good discriminative ability for many diseases. Larger data bases with standard clinical information are often available and prognostic indices exhibiting the full information from the data could be derived in a similar manner as done here. Using the NPI as an example we illustrate how to derive an index for a survival time outcome and how to assess its prognostic ability. In order to increase readability and understandability of the various analyses we will provide a technical summary of the study similar to the REMARK profile [38].

A ‘good’ predictor summarizing the standard information from clinical data can be used as a suitable benchmark to assess the added value of new information. In the context of research such a benchmark would be highly relevant for the discussion about clinical predictors, molecular predictors or combined predictors. Compared to a ‘good’ clinical predictor as an adequate benchmark it is more difficult for a molecular predictor or a combined predictor to substantially increase the discriminative ability. This will influence the role of predictors in patient management. In section 2 we introduce the data, section 3 introduces the statistical methods used to derive multivariable prognostic models and to assess the prognostic ability of a model. In section 4 we present results and propose an extended version of the NPI. Although it needs only information about hormone receptor status in addition to the three original NPI components, it has much larger discriminative ability than the NPI. With the main emphasize on the importance of a ‘standard’ prognostic index, methodological issues to derive such an index and its clinical relevance, we discuss our findings in section 5.

## Methods and Data

### Data

All patients had primary surgery for breast cancer between January 1984 and December 1998 at the surgical clinic of the Charité hospital in Berlin and were followed up until 2007. Nearly all of them were operated by the first author and he had them included in a clinical database, consisting of 2062 cases. In order to define a more homogeneous and sensible patient population for analysis, we had defined several inclusion and exclusion criteria. Accordingly, we excluded 502 patients, leaving 1560 patients for the analysis. Main reasons for exclusion were breast cancer in situ (n = 134) and age older than 80 years (n = 123), further reasons are briefly summarized in the REMARK type profile (Table 1) and details can be found in Winzer et al [28]. We use data from clinical follow-up and consider overall survival (OS) with all causes of death as an event. The number of events for OS is 221. During 1984 and 1998 treatment of patients changed severely worldwide. Winzer et al [28] provide details about treatment strategies in three periods (1984–1990, 1991–1993, 1994–1998). During the first period, most of the patients (73.4%) had a mastectomy (±) radiotherapy and did not receive adjuvant treatments. In the third period 61% had breast conserving treatment (±) radiotherapy and a systemic treatment. For details of local and adjuvant therapy, measurement techniques of prognostic factors, influence of prognostic factors on choice of treatment and further details concerning follow-up, we refer to Winzer et al [28] and cited references. To account for different treatment strategies, all Cox models were stratified according to therapy strata.

**Table 1:** REMARK type profile.

**a) Patients, treatments and variables**				
**Study and marker**	**Remarks**			
Markerhandled	M = NPI*Continuous and categorical. Cutpoints as predefined in the literature. For details see Blamey et al [27].*			
Further variables	v1 = Tumor Size, v2 = No. of pos. Lymph Nodes, v3 = Tumor Grade, v4 = Age, v5 = Histology, v6 = Hormone Receptor Status, v7 = Menopausal Status, v8 = Vessel Invasion, v9 = Lymphatic Vessel Invasion			
**Patients**	**n**	**Remarks**		
Assessed for eligibility	2062	**Disease:** Primary Breast Cancer**Patient source:** Database Surgical clinic Charité, Berlin. All patients with surgery from 1^st^ Jan. 1984 to 31^st^ Dec. 1998.		
Excluded	502	63 metastasis, 73 previous carcinoma other than breast cancer, 86 primary breast cancer prior to the study, 134 breast cancer in situ, 8 pt0, 123 older than 80 years, 20 neo-adjuvant chemotherapy, 71 death within first months of surgery, three or more standard prognostic factors missing. For some patients, more than one exclusion criterion applied.		
Included	1560	Previously untreated.***Treatment*:** *Local therapy: BCT or mastectomy with or without radiotherapy, adjuvant therapy: chemo (y/n), hormone (y/n). For details see Add file 1 and Table 2 in Winzer et al [28]*		
With outcome events	221	Overall survival: death from any cause		
**b) Statistical analyses.** All analyses using a Cox model are stratified for strata according to therapy. There are 8 strata defined by the combination of surgery, radiotherapy (y/n) and systemic therapy (y/n (no chemotherapy and no hormone therapy))				
**Analysis**	**Patients**	**Events**	**Variables considered**	**Results/ remarks**
IDA 1^1^: Imputation for missing values	1560	NR ^2^	v1(94), v2 (68), v3(217), v6(490), v7(54)	Variables (number of patients) with imputed values
A1^3^: NPI (3)	1560	221	NPI	Prognostic value of NPI in 3 categories (Table 2, Fig 1, Table 3)
A2: NPI (6)	1560	221	NPI	6 categories (Fig 1, Table 3)
C1^4^: Check of PH^5^ in NPI (3) and in NPI (6)	1560	221	NPI	Fig 2, S2 Fig and non-significant result of FPT (see last paragraph 4.2).
A3: NPIcont.	1560	221	NPI	More information from continuous data? (Table 3)
C2: NPIcont. has a linear effect	1560	221	NPI	FP2 function not significantly better, see 4.3.1
C3: Check of PH^5^ in NPIcont.	1560	221	NPI	Non-significant result of FPT (see last paragraph 4.3.1)
A4: MFP^6^ of the three NPI variables (univ. and multivariable)	1560	221	v1, v2, v3	Table 4
A5: Functional form for nodes	1560	221	v2	Fig 3
A6: Prognostic value and additional value of further variables (univ. and multiv.)	1560	221	NPI, v4, v5, v6, v7, v8, v9	Table 5, Fig 4
A7: MFP using all available information	1560	221	v1, v2, v3, v4, v5, v6, v7, v8, v9	Final MFP model in Table 6, see 4.5
A8: Measures of separation	1560	221	NPI, v1, v2, v3, v4, v5, v6, v7, v8, v9	Table 7, see 4.6
C4: Check of PH^5^ in MFP model	1560	221	v1, v2, v3, v6	Non-significant result of FPT (see end of 4.5)

REMARK type profile providing an overview of the patient population, variables in the study and analyses conducted. The Nottingham Prognostic Index (NPI) is the marker of main interest. NPI(3) and NPI(6) denote classifications into 3 and 6 prognostic groups, respectively.

^1^ Initial data analysis

^2^ not relevant

^3^ Ai–analysis no. i

^4^ Ci–check number i of an assumption

^5^ Proportional hazards assumption of the Cox model

^6^ Multivariable Fractional Polynomial procedure

The study was retrospective and we did not obtain informed consent from the patients. However, only anonymized data is used. There was no necessity to contact an Ethical Committee for this re-analysis of data. The patient records were not de-identified before entry in the original database. However, before starting the first analysis [28] KJW had received a positive vote by the authorized local data protection person for an analysis of the data base from breast cancer patients of the Charité Hospital. In addition this positive vote allows comparing follow-up data with the information given in the cancer registry and data from the residence registration office in Berlin. It is signed by Heiko Wiese and dated May 16 2012. KJW sent the database to AB to subtract the relevant information. AB created a subfile which was used in the earlier paper [28] and in this new analysis. Information specifically referring to the patient (such as name and address) was not stored. Specific dates (such as birthday or date of surgery) were only used to create relevant information such as age, survival time or year of surgery but will not be included in the publicly available file. This file will only include all variables mentioned in Table 1 for the 1560 patient included in the analysis. To destroy the chronological order of patients’ entry in the database AB randomized the order of patients’ records in the publicly available file. With these procedures we protect against identification of individual patients while allowing conducting analyses presented. KJW and AB have separate lists allowing them to restore patient identification.

The distribution of the nine standard prognostic factors and of treatment is given in S1 Table. Tumor grade has a larger percentage of missing data (13.9%) and some of the other factors have missing data in a smaller percentage of patients. In order to use all patients for the multivariable analysis we imputed missing data in two steps. In a first step, missing information was replaced based on clinical criteria (related clinical factors), if applicable. Since this strategy was not very successful, it was decided to drop all observations with more than three missing values in the standard prognostic factors (N = 2) and impute the remaining missing values using single univariable imputation sampling [39] based on observed survival times. In all analyses we will use the data after imputation for missing information (N = 1560). The distribution of the resulting NPI is given in S1 Fig. As nodes and grade both contribute with 1 to 3 points to the Nottingham Prognostic Index, the resulting distribution of the NPI has several peaks.

### Nottingham Prognostic Index

The three components lymph node stage (LN), grade and tumor size are combined to the index

NPI=LN(1−3)+Grade(1−3)+maximum diameter(cm)•0.2,

where LN is a categorized version of the number of positive lymph nodes (1 –zero positive nodes, 2 –one to three positive nodes, 3 –four or more positive nodes). In our data, this gives an observed range of NPI from 2.02 (LN negative, grade 1, 0.1 cm) to 8.2 (LN Stage 3, grade 3, size 11.0cm) Originally the two cutpoints 3.4 and 5.4 and later up to five cutpoints (2.4, 3.4, 4.4, 5.4 and 6.4) were proposed to create three to six categories with different prognosis [26,27].

Here, we will use the notation NPIcont to refer to the continuous values of NPI, NPI(3) and NPI(6) for classification schemes with three or six prognostic groups. For the three NPI(3) groups we will use the abbreviations NPI(3)-1, NPI(3)-2 and NPI(3)-3, with NPI(3)-1 denoting the group with the best prognosis. The six prognostic groups from NPI(6) will be denoted as in Blamey et al [27]: Excellent Prognostic Group (EPG) with an observed NPI range of 2.08–2.4, Good (GPG) 2.42 to 3.4; Moderate I (MPG I) 3.42 to 4.4, Moderate II (MPG II) 4.42 to 5.4, Poor (PPG) 5.42 to 6.4 and very poor (VPG) 6.42–8.2.

### The Cox Model

The Cox proportional hazard model was used to estimate the effect of variables and to derive multivariable models [29]. As any regression model, the Cox model makes several important assumptions, including proportional hazards and linear relationships between continuous covariates and the log hazard function. It is well-known that assumptions are often violated and the importance to check for them is stressed in the REMARK guidelines [38] and in many other papers and books. For studies with long-term follow-up the proportional hazard (PH) assumption is often critical, for example when a portion of patients can be considered cured after having survived for a longer period. To check the PH assumption for a categorical variable plots of log(-log survival time) versus log time may be used. They should be approximately parallel [40]. Other approaches investigate whether a Cox model with an extension for time-varying effects fit the data significantly better. Such approaches investigate for a potential interaction of a covariate with time. We will use the fractional polynomial time (FPT) algorithm [41] which was developed to detect and model potential time-varying effects with a significance level of 1%. The decision to use FPT is based on personal preferences and results of a comparison of several approaches [42].

We will use the class of fractional polynomials (FPs) to investigate whether the popular assumption of a linear effect is acceptable or whether a non-linear function improves the data fit severely and is therefore an important argument against the linearity assumption [43]. For categorical variables with an ordinal scale (such as tumor grade) we will use ordinal dummy coding and allow collapsing two neighboring categories in the model building process. For details see chapter 3.3 in [43] Royston and Sauerbrei.

For our registry data it is not possible to get unbiased estimates of effects for different treatments. However, as stressed in the REMARK guidelines (Item 10 f and Item 17) [38], it is important that different treatments are accounted for in the analysis. Details about treatment modalities and arguments to consider, type of surgery (mastectomy or breast conserving therapy) and the application of radiotherapy (yes, no) and systemic therapy (yes, no) as the three most relevant aspects of treatment given, are discussed in Winzer et al [28]. To account for therapy we stratified all analyses with the Cox regression model for the eight strata defined by the combination of the three binary variables.

### Multivariable Fractional Polynomials to Model Continuous Variables

A method for variable selection is required when a larger number of variables need to be assessed for their influence on an outcome of interest. For each continuous variable it is also necessary to estimate their influence by considering a (non-linear) functional form. Assuming a linear function or categorizing the continuous variable and using step-functions are the preferred approaches in medicine. However, severe weaknesses of both approaches have been known for a long time. For details see Box 4 of the REMARK guidelines [38]. To avoid the severest weaknesses, the class of fractional polynomials and the corresponding function selection procedure (FSP) have been proposed [43,44]. To derive a multivariable regression model, the multivariable fractional polynomial (MFP) procedure, which combines the fractional polynomial FSP with the backward elimination procedure for variable selection, has gained popularity in the health sciences. Deriving a multivariable prognostic model for patients with breast cancer, details of the procedure are given [45], key parts of the philosophy to prefer MFP to other multivariable approaches are discussed and illustrated [43,46].

In order to select a MFP model, the significance level is the key parameter to determine the number of variables included and the complexity of functional forms for continuous variables. For both parts we will use the popular 5% level, denoted as MFP(0.05, 0.05).

### Discriminative Ability of a Model

Kaplan-Meier plots are a popular and simple way to assess the discriminative ability of a classification scheme. However, such plots do not give an overall measure and it is difficult to compare several classification schemes. If a continuous score is categorized by using various cutpoints it is easily possible to present plots which can be very misleading. Differences of estimated survival rates depend heavily on the chosen cutpoint(s) and by creating smaller subgroups at both ends of a prognostic score, Kaplan-Meier plots may indicate extreme differences between subgroups. Specifically if the numbers of patients at risk are not given, such plots bear the risk of severe misinterpretation. The REMARK guidelines [38] (see item 15) explicitly state that numbers at risk should be provided for selected time points. We will present Kaplan-Meier plots for NPI(3) and NPI(6).

Based on the idea of measuring the separation between several Kaplan–Meier curves and simultaneously taking into account the relative frequencies of each category, the D-statistic was proposed [47]. It assumes that the proportional hazards assumption is not seriously violated, otherwise the use of this measure is doubtable. The D statistic can be transferred to a R^2^-type measure of explained variation. D and R^2^_D_ have been chosen because the latter ‘appear to be the best overall’ in a large simulation study comparing 17 R^2^-type measures proposed for survival models [48]. In addition we will use the c-index. It was chosen because it has been well known for a long time and it seems to be the measure used most often. However, for survival data it has some weaknesses and it is preferable to use a modified version [49].

### Software

Analyses were conducted with Stata/SE 13.1 [50] and some user-written Stata programs were used for imputation of missing values [39], calculation of explained variation [51] and Cox models with time-varying effects (stmfpt, available at https://portal.uni-freiburg.de/imbi/Royston-Sauerbrei-book).

## Results

To provide an overview of analyses conducted and reasons for them, we summarize this information in the bottom part of the REMARK type profile (Table 1).Information about the initial data analysis (IDA 1) is provided in chapter 2. As a result we have 1560 patients of which 221 died during the follow-up period. All analyses are based on this population. First we will only consider NPI (A1- A3) before trying to improve this index by using the full information from the three NPI components number of nodes, tumor size and tumor grade (A4-A5). Finally we investigate improvement of the prognostic ability by considering the added value of other standard variables and by using all available information to derive a MFP model (A6-A8). To compare the discriminative ability of NPI and various variants considered we summarize results from several measures of discrimination.

### Correlation between Factors of Interest

The correlation structure between potential prognostic variables can have a severe influence on the result of multivariable model building procedures. It is most easily summarized by giving absolute values of pairwise Spearman rank correlation coefficients. Very large correlations (≥ 0.7) are observed between NPI and no. of pos. lymph nodes (0.71), NPI and tumor grade (0.70), as well as age and menopausal status (0.73). In addition, larger (0.5–0.7) and medium sized (0.3–0.5) correlations are present for NPI and tumor size (0.59) and tumor size and no. of pos. lymph nodes (0.31), respectively. The absolute value of all other correlation coefficients is below 0.3 and we consider such values as having only a negligible influence on the result of multivariable model building.

### Discriminative Ability of the NPI Categories

**Fig 1:**
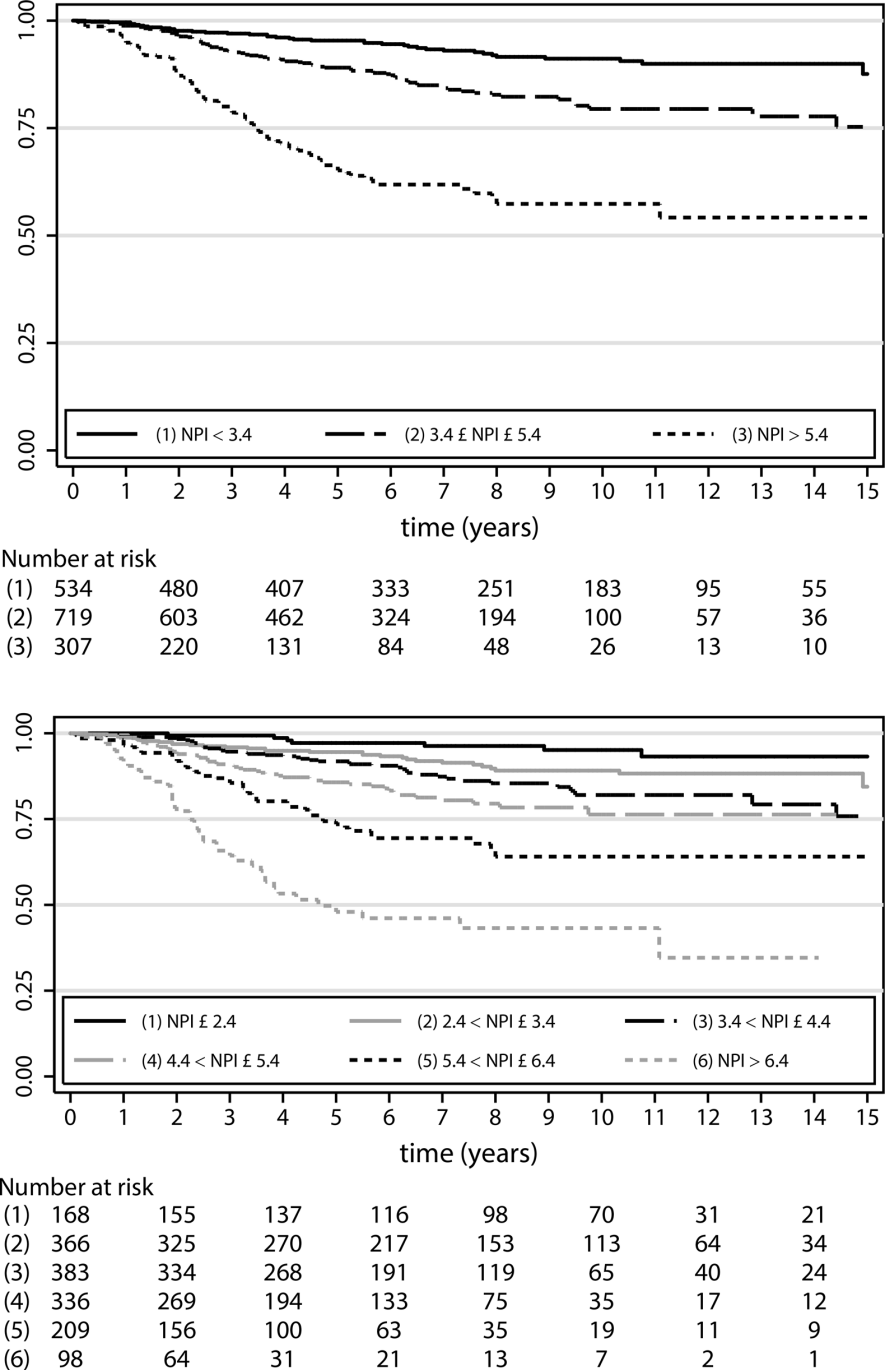
Kaplan-Meier estimates of survival probabilities for prognostic groups defined by the Nottingham Prognostic Index. Top NPI(3)– 3 groups, below NPI(6)– 6 groups.

In Fig 1 we show the survival estimates for three and six groups, respectively, derived by categorizing the NPI. With a minor exception for a short term period (up to about 2 years) even the six groups are well separated over the period of 15 years. Estimates of five-year survival rates are 95%, 89% and 65% for the three groups from NPI(3) (Table 2). NPI(6) splits each of the three groups into two separate groups and the resulting estimates (Fig 1 and S2 Table) indicate much better separation. The two most extreme groups from NPI(6) have estimated five year survival rates of 97% (EPG) and 48% (VPG), respectively. However, only a small percentage of the study population belongs to the two extreme groups (10.8%toEPG and 6.3% to VPG).

**Table 2:** Survival rates after 5, 10 and 15 years for the categories from NPI(3).

	Time	Survivor Function	Std. Error	95% CI
**NPI < 3.4**	5	0.954	0.010	[0.930, 0.969]
	10	0.912	0.015	[0.878, 0.936]
	15	0.876	0.029	[0.807, 0.921]
**3.4 ≤ NPI ≤ 5.4**	5	0.891	0.013	[0.862, 0.914]
	10	0.794	0.023	[0.745, 0.835]
	15	0.753	0.036	[0.673, 0.816]
**NPI > 5.4**	5	0.652	0.033	[0.584, 0.711]
	10	0.574	0.038	[0.495, 0.645]
	15	0.542	0.048	[0.444, 0.630]

In Table 3 we give estimates of the hazard ratios from Cox models. For NPI(3) the estimate of HR for the group with the worst prognosis (NPI > 5.4) compared to the group with the best prognosis (NPI <3.4) is 4.80. The estimated HR for the two extreme groups from NPI(6) is 14.91. These extreme categories compare the small groups with NPI < = 2.4 with NPI > 6.4. When interpreting other HR estimates given in Table 3, please note that the reference categories differ between NPI (3) and NPI(6) analyses. These results illustrate that it is often simple to derive extremely well separated subgroups and to receive large values for the estimate of the hazard ratio. Obviously, corresponding subgroups are small, confidence intervals are large and the clinical relevance of such small subgroups with an extremely good or poor prognosis is limited. This is also reflected by the small differences of the measures of separation (e.g. the C-index increases from 0.65 (NPI(3)) to 0.67 (NPI(6)) and R^2^_D_ as an overall measure derived from D increase from 0.217 to 0.245; right part of Table 3.).Hence the impression of a much better separation of NPI(6) compared to NPI(3) based on the Kaplan-Meier plots is misleading. In addition, it has to be noted that overfitting of the data is likely and results in overestimation of the difference between the two most extreme subgroups, specifically for the NPI(6) classification.

**Table 3:** Hazard ratios and discriminative ability of NPI.

	Coding	log (HR)	Hazard Ratio	95% Confidence interval of HR	R^2^_D_	C
**NPI (3 categories)**	NPI < 3.4	0	1	-	**0.217**	**0.651**
	3.4 ≤ NPI ≤ 5.4	0.54	1.72	[1.16, 2.55]		
	NPI > 5.4	1.57	4.80	[3.15, 7.30]		
**NPI (6 categories)**	NPI ≤ 2.4	0	1	0	**0.245**	**0.673**
	2.4 < NPI ≤ 3.4	0.72	2.06	[0.90, 4.71]		
	3.4 < NPI ≤ 4.4	1.00	2.72	[1.20, 6.17]		
	4.4 < NPI ≤ 5.4	1.24	3.45	[1.51, 7.87]		
	5.4 < NPI ≤ 6.4	1.84	6.32	[2.75, 14.53]		
	NPI > 6.4	2.70	14.91	[6.42, 34.66]		
**NPI continuous**^1^		0.51	1.67	[1.49, 1.86]	**0.219**	**0.675**

For three version of NPI estimated hazard ratios (left) and measures of the discriminative ability (right) of the standard CoxPH model. All analyses stratified by the eight treatment strata (see Table 1)

^1^ increase of the hazard ratio for one unit

**Fig 2:**
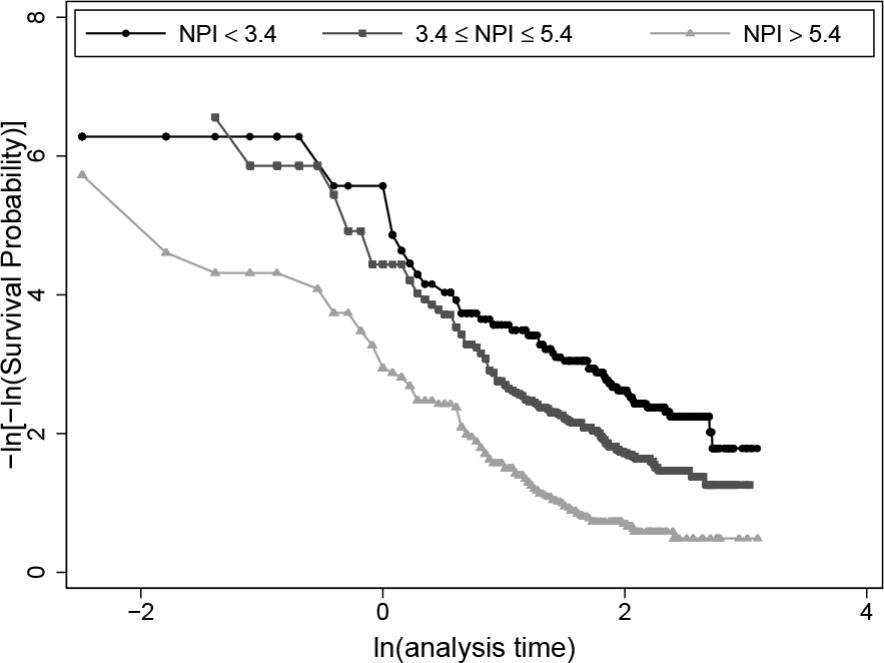
Log-log plot for NPI(3). Log-log plot to check the proportional hazards assumption of the Cox-model for three prognostic groups according to NPI(3).

Estimates of the hazard ratio and the measures D with corresponding R^2^_D_ are derived under the assumptions of the Cox proportional hazards model. For both classification schemes the log-log plots (Fig 2 for NPI (3) and S2 Fig for NPI (6)) indicate some minor violations of the PH assumption at the beginning of the follow-up time. Checking for non-proportionality by using the fractional polynomial time (FPT) algorithm did not exhibit a significant time-varying effect. P values of the test for non-proportionality (FP2 function vs. PH) were 0.0647 for NPI (3) and 0.343 for NPI (6), respectively. Altogether we conclude that the PH assumption is acceptable.

### Extensions of NPI Categories by Using Full Information

#### Continuous NPI

To avoid problems caused by categorization it may be more suitable to provide estimates for the continuous variable NPI. The estimated increase in risk for one unit (e.g. from 3.13 to 4.13 or from 5.09 to 6.09) is 1.67 (see last line of Table 3). Using NPI as a continuous variable hardly influences the measures for discrimination.

Estimating the effect for the continuous variable NPI we have assumed that a linear function describes the influence of NPI on our outcome. Using the MFP function selection procedure and a 5% significance level we have checked that non-linear functions do not improve the fit and that the linearity assumption is acceptable. The test of the proportional hazards assumption did not exhibit any significant violations.

#### MFP of the three components

The NPI consists of the three components: tumor size, no of positive lymph nodes and tumor grade. In the original analysis node involvement was categorized and three groups were considered. The relative weight of each component was estimated as given in the Introduction. Here, we consider a possible improvement of the prognostic ability by (i) using the full information on the number of positive nodes, (ii) allowing non-linear functions to estimate the effect of the two continuous variable and (iii) re-estimating the relative weights of each of the three variables. In Table 4 we give estimates of the three components in univariable Cox models assuming a linear effect for tumor size and the number of positive nodes. Results of the FP analyses show that non-linear functions describe the functional relationship with the outcome better (in terms of the deviance). The Multivariable FP (MFP) approach selects a model with a linear effect for tumor size, a non-linear effect for the number of nodes and combines categories 1 and 2 for grade, estimating an increased risk for grade 3 compared to the others.

**Table 4:** Estimated effects of NPI components.

		Univariable				Multivariable	
		Linear function		FP function		MFP model	
	Coding	HR	95% CI	HR	95% CI	HR	95% CI
**Tumor size**	in cm (linear)	1.18	[1.11, 1.25]			1.11	[1.04, 1.19]
	(size)^1/2^			2.06	[1.58, 2.68]		
**No. of pos. lymph nodes**	linear	1.08	[1.06, 1.09]				
	log(nodes+1)			1.90	[1.65, 2.20]		
	(nodes+1)^-1/2^					0.12	[0.07, 0.21]
**Tumor grade**	1	1	-	not relevant		1	-
	2	1.72	[1.12, 2.64]	not relevant		1	-
	3	2.34	[1.52, 3.60]	not relevant		1.44	[1.09, 1.91]

Univariable and multivariable Cox PH models assuming a linear effect for continuous variables or choosing the functional form by using the function selection procedure of the fractional polynomial approach. The significance level for both variables and functions is 0.05. All analyses stratified by the eight treatment strata (see Table 1)

**Fig 3:**
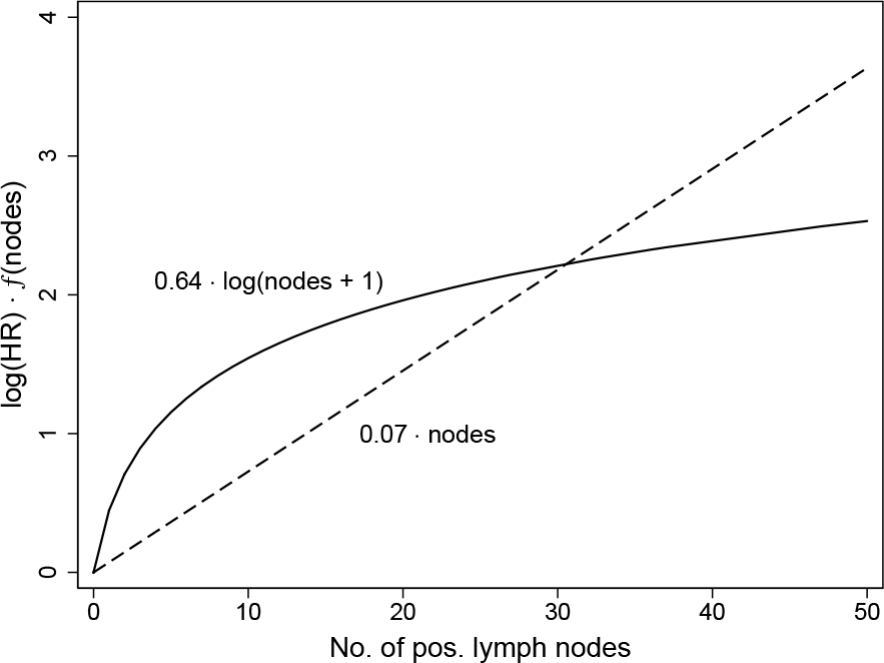
Functional form for no. of positive nodes for two models. Predictor for univariable analysis of no. of positive lymph nodes derived by two Cox models assuming a linear effect (dashed line) or the significant improvement from the class of FP models (solid line).

For univariable analyses Fig 3 illustrates the difference between the popular approach to assume a linear functional relationship for a continuous variable and the fractional polynomial approach which checks whether a non-linear function from the FP class fits the data significantly better. Differences indicate that models assuming a linear functional relationship underestimate the risk for very low values such as 2 positive nodes or a tumor size of 5 mm and overestimate the risk (severely) for very large values such as 30 positive nodes or a tumor size of 100 mm.

### Univariable Assessment of the Added Value of Other Standard Factors

In addition to the three NPI components there are six additional standard factors available. In Table 5 we give estimates of their effects in a univariable model. Age, menopausal status and histology have no influence on survival time, whereas the effects of the three binary variables hormone receptor status, vessel invasion and lymphatic vessel invasion are significant at the 5% level. The effect of hormone receptor status is strongest and it is the only effect remaining significant after adjustment for NPIcont. These results indicate that hormone receptor status is the only of the six variables which has a significant effect in addition to NPI.

**Table 5:** Estimated effects of additional variables.

		Univariable model				Multivariable model	
		Unadjusted		Adjusted for NPI*			
	Coding	HR	95% CI	HR	95% CI	HR	95% CI
**NPI**	continuous	1.67	[1.49, 1.86]	*	*	1.57	[1.39, 1.77]
**Age**	continuous	1.00	[0.99, 1.01]	1.00	[0.99, 1.01]	0.99	[0.98, 1.01]
**Histology**	invasive ductal	1	-	1	-	1	-
	invasive lobular	0.88	[0.53, 1.44]	0.91	[0.55, 1.49]	0.91	[0.55, 1.50]
	other	0.64	[0.34, 1.20]	0.74	[0.39, 1.40]	0.66	[0.35, 1.26]
**Hormone receptor status**	negative	1	-	1	-	1	-
	positive	0.41	[0.32, 0.54]	0.49	[0.37, 0.64]	0.48	[0.37, 0.63]
**Meno- pausal status**	pre-menopausal	1	-	1	-	1	-
	post-menopausal	1.05	[0.78, 1.41]	1.14	[0.85, 1.53]	1.35	[0.89, 2.04]
**Vessel invasion**	V0	1	-	1	-	1	-
	V1	1.79	[1.15, 2.78]	1.22	[0.78, 1.92]	1.10	[0.69, 1.76]
**Lymphatic vessel invasion**	L0	1	-	1	-	1	-
	L1	1.62	[1.22, 2.17]	1.24	[0.93, 1.66]	1.21	[0.89, 1.64]

Univariable (without and with adjustment) and multivariable (‘full’) CoxPH models. For continuous variables a linear effect is assumed. All analyses stratified by the eight treatment strata (see Table 1)

* Estimates for effects of NPI vary slightly (1.60–1.67) for the 6 different models.

### Multivariable Models

The multivariable model given in Table 5 includes NPI and the six additional factors, assuming linear effects for continuous variables. For an MFP analysis it is the ‘starting’ model and the algorithm investigates simultaneously whether any of the variables can be eliminated and whether any of the continuous variables have a non-linear effect. This ‘full’ model includes several variables with a ‘weak’ or no effect and only NPIcont and hormone receptor status are significant. Using MFP(0.05, 0.05) to select a simpler model, only NPIcont and hormone receptor status remain in the model.For NPI a linear function is chosen and the estimated effect is 1.60 (1.43–1.80) per unit increase for NPI. This simple model estimates that hormone receptor positive tumors have a severely decreased risk of 0.49 (0.37–0.64) compared to receptor negative tumors.

**Fig 4:**
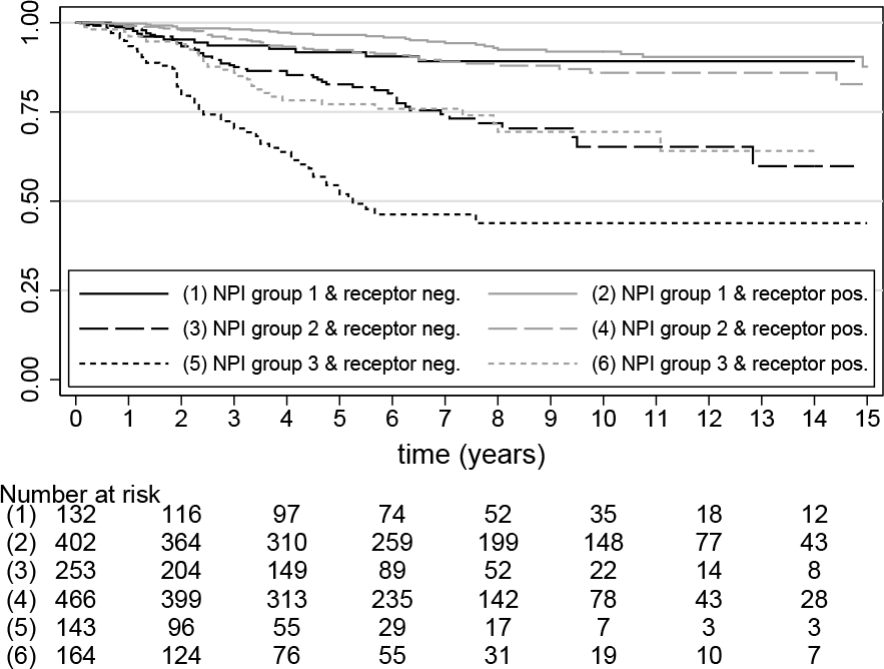
Kaplan-Meier curve for combinations of NPI(3) and hormone receptor. NPI groups are defined as 1: NPI<3.4; 2: 3.4≤NPI≤5.4; 3: NPI>5.4.

In Fig 4 we give Kaplan-Meier estimates of survival rates for the six groups derived by a combination of NPI(3) and receptor status (see also S3 Table). This figure illustrates that receptor status has a strong influence on the survival rate for patients in the worst NPI group (10 year survival rates for receptor positive are 0.69 whereas the corresponding value for receptor negative patients is 0.44). Over the full observation period survival rates of receptor positive patients from NPI(3)-3 are very similar to survival rates of receptor negative patients from NPI(3)-2. For patients from the NPI(3)-2 group hormone receptor status has also a strong influence on the survival rate (10 year rate is 0.86 for pos and 0.65 for neg), whereas the hormone receptor effect is small for NPI(3)-1 patients. Estimates at 10 years are 0.92 (pos), 0.89 (neg). For the NPI(3)-1 group the effect of hormone receptor status seems to vary slightly in time.

To use all information from the nine available variables we repeat the MFP analysis from above, but consider the three NPI components separately. This analysis would also allow eliminating one or more of the NPI components if they have no significant effect in a multivariable context. However, all three components remain and hormone receptor is added in the final model. We propose to use the term extended Nottingham Prognostic Index for this resulting model and define

NPIext=0.108*tumor size(cm)+0.543*log(no. pos. nodes+1)+0.404*Ind(if tumor grade is2or3)-0.766*Ind(if hormone receptor is positive),

where Ind is the indicator function taking the values 0 (condition is not fulfilled) or 1 (condition is fulfilled).

Compared to NPI it adds hormone receptor status as a binary variable, estimates a non-linear functional influence of the number of positive lymph nodes and weighs the relative influence of the variables differently (Table 6). The results broadly coincide with the MFP model for the three NPI components (see Table 4), but the inclusion of hormone receptor status has slightly changed the functional form for the number of nodes. Please note that the chosen power terms are different (-0.5 in Table 4 and 0 (log function) in Table 6), implying that parameter estimates are very different, but the corresponding functions are very similar (not presented). The addition of hormone receptor status seems to have the most important influence on the effect of tumor grading. In the model in Table 4 grade 1 and 2 were collapsed (meaning that the prognosis of these two categories is identical and only grade 3 has a worse prognosis) whereas grade 2 was collapsed with 3 in the new model (grade 1 has a better prognosis than the others; see Table 6).

**Table 6:** MFP model.

	Coding	log (HR)	Hazard Ratio	95% Confidence interval of HR
**Tumor size**	in cm (linear)	0.11	1.11	[1.04, 1.19]
**No. of pos. lymph nodes**	log(nodes+1)	0.54	1.72	[1.48, 1.99]
**Tumor grade**	1	0	1	-
	2 and 3	0.40	1.50	[0.99, 2.26]
**Hormone receptor**	negative	0	1	-
	positive	-0.77	0.46	[0.35, 0.61]

MFP(0.05, 0.05) model for the three individual components of NPI and the six variables v4-v9. Five of these variables are eliminated from the final model. Analysis stratified by the eight treatment strata (see Table 1)

### Comparison of Models

To compare performance of various models we give estimates of the C-index, the D-measure and corresponding R^2^_D_ for the major’ models considered in the paper. Compared to NPI(3) our final MFP model improves the C-index from 0.651 to 0.714. This notable improvement is a result of several minor improvements starting with the use of the full NPI information instead of categorization with three categories, some improvement by using the full information from the number of positive nodes and the strongest increase comes from adding hormone receptor status. A similar improvement is indicated by the D-measure and R^2^_D_. An improved R^2^ value from 0.217 to 0.290 is substantial (34%), but may to some extent be caused by overfitting the data. The potential problem of overfitting does not apply to the three models solely depending on the NPI. By using resampling methods it would be possible to (partly) correct for overoptimism, but that is beyond the scope of this paper. Further investigations in a related methodological paper will allow deriving estimates for the performance measures which are less prone to overoptimism.

## Discussion

In a paper written in a time before the era of genetic information had really started, Sauerbrei et al [45] stressed the importance of an accepted prognostic model based on standard factors: ‘*An important step for an improvement in summarizing information from prognostic factors would be a widely accepted standard prognostic model. We see the Nottingham Prognostic Index as the most often validated and accepted classification scheme. As recently published for node-negative patients where, in addition to changes to the weights of the component of the NPI, in one of our proposals only age was added to the NPI [24], we see the results from the NPI and our proposals as a starting point for an urgently needed accepted and sensible description of the influence of standard factors.*’ At that time in breast cancer the prognostic effect of more than 100 factors was discussed controversially and the idea was to have a standard model and to check whether a new factor improved the separation of prognostic groups derived from a prognostic model. At least, a standard model would have helped to standardize analyses across studies and to avoid many claims concerning the relevance of a new prognostic factor. Nowadays, there is still a controversial discussion concerning factors such as Her2-neu and Ki-67 [52] and since the intensive research on molecular data, the necessity for a ‘good’ predictive model based on ‘standard’ factors has even increased, at least when clinical usefulness is considered an important criterion.

### Use and Usefulness of the NPI

The NPI was developed more than 30 years ago using standard information and (from today’s point of view) a simple approach to derive a Cox model. It is very well validated in many studies worldwide and oftentimes used in patient care. Beside of some disagreement with point 4, we fully agree with the synopsis concerning the usefulness of prognostic indices from a group of authors from Nottingham, some of which belong to the original developers of the NPI [27]. Blamey et al state

‘*The NPI has satisfied the criteria which should be applied to all claimed methods for prognostic prediction*, *namely ability*

1. *To separate patients into groups with significantly differing survival chances*.
2. *To achieve wide separation*, *i*.*e*. *to recognise a ‘cured’ group and a group with poor survival*.
3. *To place a sufficient percentage of cases into each group*.
4. *To be applicable to all operable breast cancers*, *i*.*e*. *small*, *screen detected as well as symptomatic and those in patients of young age*.
5. *To have been prospectively validated intra-centre in a new tumour set from that on which it was derived and intercentre and internationally*.
6. *To be capable of measurement in all units and inexpensive*.*’*

Because NPI satisfies the most important criteria for a sensible and useful prognostic index, we consider it as a suitable starting point when trying to derive an improved classification scheme. Improvement has to be judged by the added value related to a suitable measure of prognostic separation.

### Potential Improvements of the NPI

Deriving a very simple index with few points and using a simple classification scheme was important for practical use in the last century. Measures indicate improved separation by extending the original proposal with three categories (NPI(3)) to six categories (NPI(6)). However, the weights of the three components tumor size, number of positive nodes and tumor grade remained unchanged, categorization disregards parts of the relevant information from continuous data and finally no further factor was added to the NPI in later research.

As in many studies before, the prognostic groups from NPI(3) and NPI(6) separate our patient population well. However, it is well known that the number of positive lymph nodes is a prognostic factor with a very strong effect and the NPI uses only a simple classification into three groups, therefore ignoring a lot of the information from this marker. For example, the NPI uses the same weight for patients with four and with 25 positive lymph nodes. It is becoming more and more accepted that categorization has severe weaknesses when developing a multivariable model and that continuous variables should be modelled in a suitable way [45]. Categorization is required for medical decision making but it has severe weakness when deriving multivariable models. Categorization should be postponed to the final step of developing a prognostic classification scheme.

By using the full information from the continuous variables tumor size and number of positive lymph nodes and by adding hormone receptor status, we could improve the prognostic ability. Compared to many other studies investigating the value of new factors added to the NPI, our ‘added value’ from the better use of standard factors is much larger. By using three suitable measures of separation we summarize the information from all ‘major’ models in Table 7. It is well known that scores derived in a data dependent way may be too optimistic and need validation in independent data [24,36,38]. As all components from our new score are generally available, validation studies can be easily conducted.

**Table 7:** Summary of ‘major’ models and corresponding measures of separation.

Prognostic factors	D	R^2^_D_	C
NPI with 3 categories	1.077	0.217	0.651
NPI with 6 categories	1.165	0.245	0.673
NPI continuous	1.084	0.219	0.675
Tumor size, No. of pos. lymph nodes (transformed), Tumor grade (3 vs. 1/2)	1.158	0.243	0.677
NPI, Age, Histology, Hormone receptor status, Menopausal status, Vessel invasion,Lymphatic vessel invasion	1.268	0.277	0.706
NPI, Hormone receptor status	1.231	0.266	0.707
Tumor size, No. of pos. lymph nodes (transformed), Tumor grade (2/3 vs. 1), Hormone receptor status	1.308	0.290	0.714

While we concentrated on a more suitable modelling of the data, using a two-step approach to try to improve the prognostic ability of a score is also possible. Rakha et al [53] started by using multivariable clustering techniques to identify seven key molecular classes. Within each class they estimate the effect of several clinicopathological factors in a multivariable Cox model. Simply by chance the size of the estimate effects varies and a specific factor (say tumor size) gets a large effect in some of the molecular classes and a small effect in others. For each molecular class this gives a separate (NPI-type) formula and this complex classification scheme is called Nottingham Prognostic Index Plus (NPI+). In principle, NPI+ is a very complex index proposing interactions between the molecular classes and the clinicopathological factors. Unfortunately it is not possible to assess how much NPI+ improves on NPI(3) or NPI(6) as presentation of results is restricted to Kaplan-Meier plots for survival time. Measures of the discriminative ability of the indices are not given.

For many years NPI(3) has been used for decision making in clinical practice. Barton et al [52] investigated for agreement between risk categorizations by NPI(3) and the IHC4+C score (also categorized into 3 groups). Risk classifications (low, medium, high) agreed in 60.4%. Studies with access to relevant data of the IHC4+C score could check whether our modified NPI index agrees better with the IHC4+C score, specifically differences in the NPI(medium) category are severe. However, differences in risk classifications do not directly imply different treatments for patients. The comparisons of treatments require randomized trials and the importance of risk classifications for treatment decisions needs investigations in relevant subgroups and corresponding tests for interaction with treatment.

### Methodological Issues

Compared to the methodology for multivariable model building at the time the NPI was developed, statistical methodology has seen severe improvements. Unfortunately, many of these developments are ignored in practice [54]. In addition, reporting of many studies in the health sciences is insufficient and incomplete [36,38], making the assessment of results difficult. By providing a REMARK type profile [38] we give details about our patient population, illustrate all steps of our model development and assessment and refer to some highly relevant checks of important assumptions of the Cox model. Unfortunately, there are no generally accepted state-of-the-art analyses and measures [54]. For many years some of us have worked in this area and we used our preferred approaches. We are well aware that the results are too optimistic if model development and model assessment are based on the same data. Therefore we expect that the added value of our new proposal is slightly overestimated. This and several other relevant methodological issues will be discussed in a follow-up paper.

### Summary

Using improved statistical methodology and generally available standard information we propose to slightly extend the NPI. Using the full information from continuous variables we derive the index

NPIext=0.108*tumor size(cm)+0.543*log(no. pos. nodes+1)+0.404*Ind(if tumor grade is2or3)-0.766*Ind(if hormone receptor is positive)

which leads to an important increase in its prognostic ability and a related classification scheme can be easily derived and generally used for patient handling.

Most of the statistical issues illustrated in the context of breast cancer and the Nottingham Prognostic Index apply in a similar manner to other projects aiming to derive a prognostic index for a survival time outcome. To better illustrate which analyses and checks of assumptions are relevant we summarize all steps conducted in a REMARK type profile (Altman, [38]). We propose to use such an approach to derive a suitable measure summarizing the information from standard factors in other diseases.

## Conclusion

Using the well-established NPI as an example, we show that it is possible to derive an extended index NPIext with substantially improved prognostic ability. Using more sophisticated statistical methodology we propose to weight tumor size, no of positive nodes and tumor grade differently and to add hormone receptor status. As all four prognostic markers are available for most breast cancer patients, it can be used for patient management worldwide. For prognostic research in breast cancer, it can be used to summarize standard clinical information, also serving as a benchmark to assess the added value of new clinical or molecular markers in single studies as well as in the assessment of a marker or a genomic signature in meta-analysis. Statistical methods used are generally available and can be used for similar analyses in other diseases.

## Supporting Information

S1 Dataset (XLSX)

S1 Fig Distribution of the Nottingham Prognostic Index. (PDF)

S2 Fig Log-log plot to check the proportional hazards assumption of the Cox-model for six prognostic groups according to NPI(6). (PDF)

S1 Table Distribution of prognostic factors and treatment (n = 1560 patients). (PDF)

S2 Table Survival rates after five and ten years for the six NPI categories. (PDF)

S3 Table Estimated survival time for groups defined by the combination of NPI(3) and hormone receptor status. (PDF)
