# Supplementary figures and images for: Improving the Prognostic Ability through Better Use of Standard Clinical Data - The Nottingham Prognostic Index as an Example
Source: PLoS One. 2016 Mar 3;11(3):e0149977. doi: 10.1371/journal.pone.0149977 (PMC4777365; doi:10.1371/journal.pone.0149977)

**S1 Fig.** Distribution of the Nottingham Prognostic Index

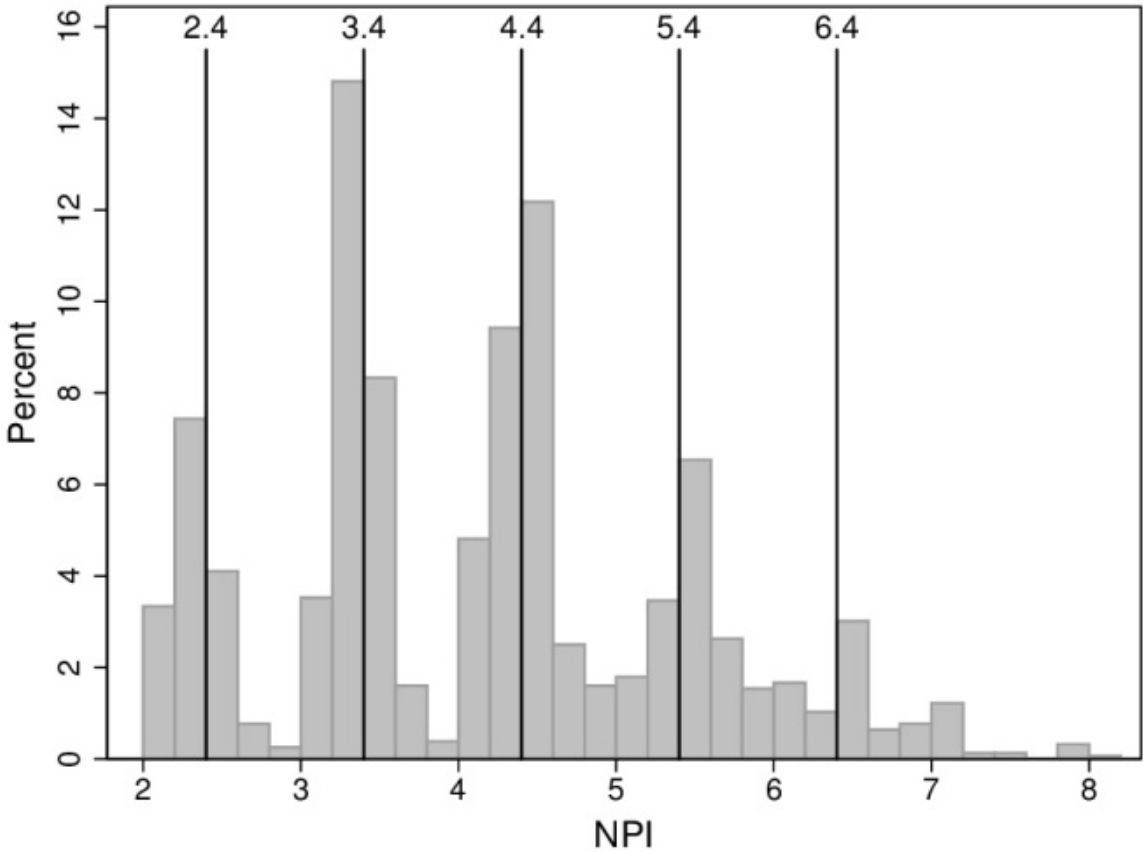

Supplement: S1 Fig — (PDF) [file pone.0149977.s002.pdf]
