# Supplementary material for: Improving the Prognostic Ability through Better Use of Standard Clinical Data - The Nottingham Prognostic Index as an Example
Source: PLoS One. 2016 Mar 3;11(3):e0149977. doi: 10.1371/journal.pone.0149977 (PMC4777365; doi:10.1371/journal.pone.0149977)

S2 Fig. Log-log plot to check the proportional hazards assumption of the Cox-model for six prognostic groups according to NPI(6)

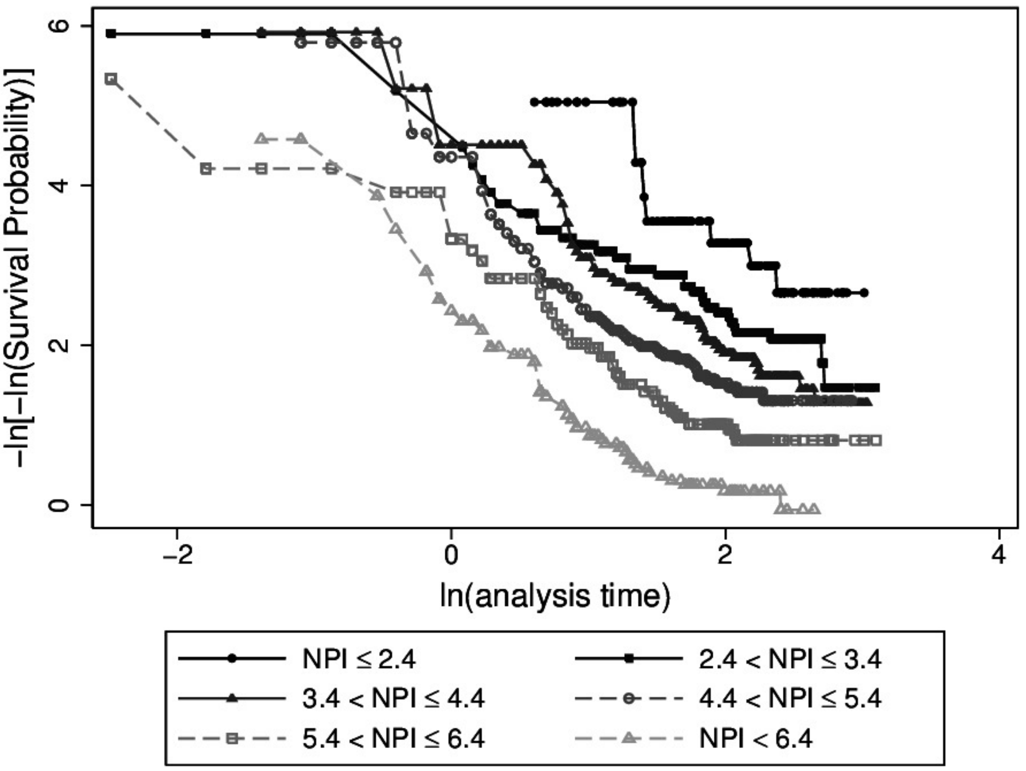

Supplement: S2 Fig — (PDF) [file pone.0149977.s003.pdf]
