# Supplementary material for: Improving the Prognostic Ability through Better Use of Standard Clinical Data - The Nottingham Prognostic Index as an Example
Source: PLoS One. 2016 Mar 3;11(3):e0149977. doi: 10.1371/journal.pone.0149977 (PMC4777365; doi:10.1371/journal.pone.0149977)
Supplement: S1 Table — (PDF) [file pone.0149977.s004.pdf]

S1 Table. Distribution of prognostic factors and treatment (n = 1560 patients)

		Coding		As measured[1]	After imputation
**Tumor size**		≤ 20mm		810 (55.3)	859 (55.1)
		21-50mm		592 (40.4)	628 (40.3)
		> 50mm		64 (4.4)	73 (4.7)
		missing		94	-
**Number of positive lymph nodes**		0		919 (61.6)	941 (60.3)
		1-3		317 (21.3)	343 (22.0)
		4-10		168 (11.3)	178 (11.4)
		> 10		88 (5.9)	98 (6.3)
		missing		68	-
**Grade**		G1		291 (21.7)	346 (22.2)
		G2		610 (45.4)	693 (44.4)
		G3		442 (32.9)	521 (33.4)
		missing		217	-
**Age**		≤ 40		106 (6.8)	106 (6.8)
		41 – 50		325 (20.8)	325 (20.8)
		51 – 60		463 (29.7)	463 (29.7)
		61 – 70		388 (24.9)	388 (24.9)
		> 70		278 (17.8)	278 (17.8)
**Menopausal status**		pre-menopausal		438 (29.1)	449 (28.8)
		post-menopausal		1,068 (70.9)	1,111 (71.2)
		missing		54	-
**Hormone receptor status**		negative		367 (34.3)	528 (33.9)
		positive		703 (65.7)	1,032 (66.2)
		missing		490	-
**Histology**		invasive ductal		1,321 (84.7)	1,321 (84.7)
		invasive lobular		137 (8.8)	137 (8.8)
		other		102 (6.5)	102 (6.5)
**Vessel invasion**		V0		1,487 (95.3)	1,487 (95.3)
		V1		73 (4.7)	73 (4.7)
**Lymphatic vessel invasion**		L0		1,200 (76.9)	1,200 (76.9)
		L1		360 (23.1)	360 (23.1)
**Treatment**	**Mastec-tomy**	no RT	no syst	365 (23.7)	370 (23.7)
		no RT	syst[2]	286 (18.6)	289 (18.5)
		RT	no syst	303 (19.7)	310 (19.9)
		RT	syst	62 (4.0)	62 (4.0)
		missing		15	-
	**BCT[3]**	no RT	no syst	58 (3.8)	58 (3.7)
		no RT	syst	69 (4.5)	70 (4.5)
		RT	no syst	212 (13.8)	213 (13.7)
		RT	syst	187 (12.1)	188 (12.1)
		missing		3	-

[1] percentages relative to non-missing values;

[2] systemic treatment with chemotherapy, hormone therapy or both;

[3] breast-conserving therapy
