# Supplementary material for: Improving the Prognostic Ability through Better Use of Standard Clinical Data - The Nottingham Prognostic Index as an Example
Source: PLoS One. 2016 Mar 3;11(3):e0149977. doi: 10.1371/journal.pone.0149977 (PMC4777365; doi:10.1371/journal.pone.0149977)
Supplement: S2 Table — (PDF) [file pone.0149977.s005.pdf]

S2 Table. Survival rates after 5 and 10 years for the 6 NPI categories.

	Time	Survivor Function	Std. Error	95% CI
NPI ≤ 2.4	5	0.972	0.014	0.926, 0.989
	10	0.951	0.020	0.893, 0.978
2.4 < NPI ≤ 3.4	5	0.945	0.013	0.915, 0.965
	10	0.891	0.020	0.845, 0.924
3.4 < NPI ≤ 4.4	5	0.918	0.016	0.882, 0.944
	10	0.820	0.029	0.754, 0.870
4.4 < NPI ≤ 5.4	5	0.857	0.022	0.808, 0.894
	10	0.763	0.036	0.684, 0.825
5.4 < NPI ≤ 6.4	5	0.735	0.037	0.654, 0.800
	10	0.641	0.048	0.539, 0.726
NPI > 6.4	5	0.480	0.060	0.359, 0.591
	10	0.432	0.063	0.307, 0.551
