# Supplementary material for: Improving the Prognostic Ability through Better Use of Standard Clinical Data - The Nottingham Prognostic Index as an Example
Source: PLoS One. 2016 Mar 3;11(3):e0149977. doi: 10.1371/journal.pone.0149977 (PMC4777365; doi:10.1371/journal.pone.0149977)
Supplement: S3 Table — (PDF) [file pone.0149977.s006.pdf]

S3 Table. Estimated survival time for groups defined by the combination of NPI(3) and hormone receptor status. Based on similarity of survival rates we have combined the six groups into three groups (lower part).

	Time	Survivor Function	Std. Error	95% CI
**NPI(3) = 1 rec. neg.**	5	0.917	0.025	0.851,0.955
	10	0.892	0.030	0.816, 0.938
**NPI(3) = 1 rec pos.**	5	0.966	0.010	0.941, 0.981
	10	0.919	0.017	0.879, 0.946
**NPI(3) = 2 rec. neg.**	5	0.827	0.027	0.767, 0.874
	10	0.652	0.052	0.541, 0.743
**NPI(3) = 2 rec. pos.**	5	0.924	0.014	0.892, 0.946
	10	0.860	0.023	0.807, 0.899
**NPI(3) = 3 rec. neg.**	5	0.519	0.051	0.416, 0.613
	10	0.438	0.055	0.329, 0.542
**NPI(3) = 3 rec. pos.**	5	0.772	0.039	0.684, 0.838
	10	0.694	0.052	0.581, 0.783

	Time	Survivor Function	Std. Error	95% CI
**All others**	5	0.940	0.008	0.922, 0.954
	10	0.891	0.013	0.863, 0.913
**NPI(3) = 2, neg. and NPI(3) = 3, pos.**	5	0.807	0.022	0.759, 0.847
	10	0.675	0.036	0.599, 0.740
**NPI(3) = 3 rec. neg.**	5	0.519	0.051	0.416, 0.613
	10	0.438	0.055	0.329, 0.542
